# 2,2′-(1-Phenyl-1*H*-pyrazole-3,5-di­yl)diphenol

**DOI:** 10.1107/S1600536809001226

**Published:** 2009-01-23

**Authors:** Sumeera Ikram, Muhammad Zia ul Haq, Amir Badshah, Aurangzeb Hasan, Michael Bolte

**Affiliations:** a Department of Chemistry, Quaid-i-Azam University, Islamabad 45320, Pakistan; b Institut für Anorganische Chemie, J. W. Goethe-Universität, Max-von-Laue-Strasse 7, 60438 Frankfurt/Main, Germany

## Abstract

The title compound, C_21_H_16_N_2_O_2_, was derived from 1-(2-hydroxy­phen­yl)-3-(-methoxy­phen­yl)propane-1,3-dione. The mol­ecular structure of the title compound is stabilized by an intra­molecular O—H⋯N hydrogen bond. The dihedral angle between the hydroxy­phenyl ring involved in this intra­molecular hydrogen bond and the pyrazole ring is significantly smaller [10.07 (6)°] than the dihedral angle between the pyrazole and the other hydroxy­phenyl ring [36.64 (5)°]. The benzene ring makes a dihedral angle of 54.95 (3)° with the pyrazole ring. The crystal packing is stabilized by O—H⋯O and O—H⋯N hydrogen bonds.

## Related literature

For the biological activity of pyrazoles, see: Beeam *et al.* (1984 ▶). For the preparation of new materials for medicine, see: Elguero (1983 ▶). For the coordination chemistry of pyrazoles, see: Bonati (1980 ▶). For their use as analytical reagents, see: Freyer & Radeglia (1981 ▶). For the synthesis of 1-(2′-hydroxy­phen­yl)-3-(2′′-methoxy­phen­yl)propane-1,3-dione, see: Ahmad *et al.* (1997 ▶).
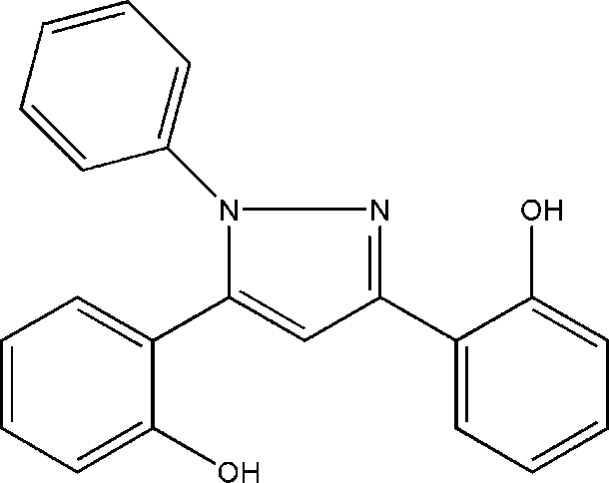


## Experimental

### 

#### Crystal data

C_21_H_16_N_2_O_2_
*M* *_r_* = 328.36
Monoclinic, 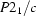

*a* = 9.7034 (8) Å
*b* = 11.7407 (9) Å
*c* = 14.9486 (14) Å
β = 104.294 (7)°
*V* = 1650.3 (2) Å^3^
*Z* = 4
Mo *K*α radiation
μ = 0.09 mm^−1^
*T* = 173 (2) K
0.48 × 0.46 × 0.46 mm

#### Data collection

Stoe IPDSII two-circle diffractometer
Absorption correction: none
12165 measured reflections
3799 independent reflections
3235 reflections with *I* > 2σ(*I*)
*R*_int_ = 0.034

#### Refinement

*R*[*F* ^2^ > 2σ(*F* ^2^)] = 0.039
*wR*(*F* ^2^) = 0.101
*S* = 1.03
3799 reflections
235 parameters
H atoms treated by a mixture of independent and constrained refinement
Δρ_max_ = 0.24 e Å^−3^
Δρ_min_ = −0.15 e Å^−3^

### 

Data collection: *X-AREA* (Stoe & Cie, 2001 ▶); cell refinement: *X-AREA*; data reduction: *X-AREA*; program(s) used to solve structure: *SHELXS97* (Sheldrick, 2008 ▶); program(s) used to refine structure: *SHELXL97* (Sheldrick, 2008 ▶); molecular graphics: *PLATON* (Spek, 2003 ▶) and *XP* in *SHELXTL-Plus* (Sheldrick, 2008 ▶); software used to prepare material for publication: *SHELXL97*.

## Supplementary Material

Crystal structure: contains datablocks I, global. DOI: 10.1107/S1600536809001226/bx2193sup1.cif

Structure factors: contains datablocks I. DOI: 10.1107/S1600536809001226/bx2193Isup2.hkl

Additional supplementary materials:  crystallographic information; 3D view; checkCIF report

## Figures and Tables

**Table 1:** Hydrogen-bond geometry (Å, °)

*D*—H⋯*A*	*D*—H	H⋯*A*	*D*⋯*A*	*D*—H⋯*A*
O2—H2⋯O1^i^	0.94 (2)	1.81 (2)	2.7524 (12)	176.6 (19)
O1—H1⋯N2	0.947 (19)	1.718 (19)	2.5863 (12)	150.9 (17)

Symmetry code: (i) 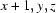.

**Fig. 1.**
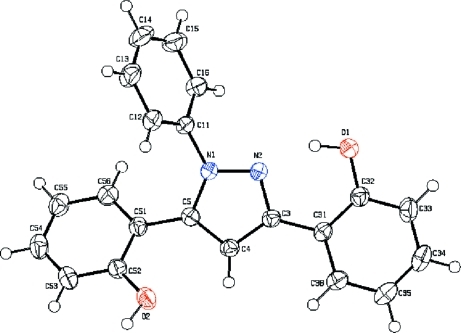
Molecular structure of the title compound. Displacement ellipsoids are drawn at the 50% probability level.

**Fig. 2.**
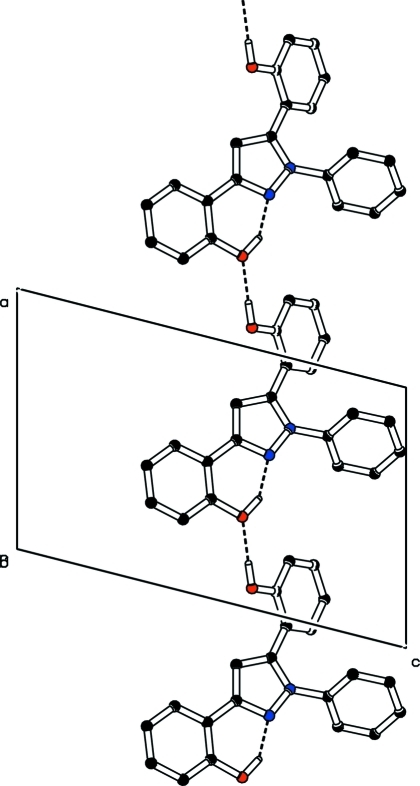
Part of the crystal structure of(I) showing the formation of a one-dimensional chain along [100] direction and the hydrogen-bonding and O-H···N intramolecular contact.

**Fig. 3.**
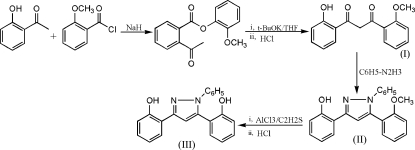
Preparation of the title compound.

## supplementary crystallographic information

### Comment

Pyrazoles are important because of their potential for biological activity. They have antipuretic, anti-inflammatory and antirheumatic effects (Beeam *et al.*, 1984). Both traditional and new scientific methods have been used to prepare new materials for medicine (Elguero *et al.*, 1983) and agriculture (Trofimenko, 1972). Neutral and anionic pyrazoles are excellent ligands and their co-ordination chemistry has been extensively studied (Bonati, 1980). Pyrazoles are also used as analytical reagents (Freyer *et al.*, 1981) The molecular structure of the title compound is stabilized by an intramolecular O-H···N hydrogen bond. The dihedral angle between the hydroxyphenyl ring involved in this intramolecular hydrogen bond is significantly smaller [10.07 (6)°] than the dihedral angle between the pyrazole and the other hydroxyphenyl ring [36.64 (5)°]. The phenyl ring makes a makes dihedral angle of 54.95 (3)° with the pyrazol ring. The crystal packing is stabilized by O-H···O hydrogen bonds.

### Experimental

1-(2'-hydroxyphenyl)-3-(2"-methoxyphenyl) propane-1,3-dione (I) was prepared by a modified Baker Venkataram rearrangement as reported earlier (Ahmad *et al.* 1997). 1-Phenyl-3,5-bis(2'-hydroxy phenyl)phyrazole(III) was synthesized by demethylation of 2-(5-(2-methoxyphenyl)-1-phenyl-1*H*-pyrazol-3-yl)phenol(II), which was prepared by refluxing 1-(2'-hydroxyphenyl)-3-(2"-methoxyphenyl) propane-1,3-dione (2.7 g, 10 mmol) with phenyl hydrazine (1.08 g,0.99 ml, 10 mmol) in 100 ml absolute ethanol for seven hours as shown in Fig. 3. The product was recrystallized using absolute ethanol. (yield: 90%, m.p: 473k)

### Refinement

H atoms bonded to C were geometrically positioned and refined using a riding model with fixed individual displacement parameters [U(H) = 1.2 *U*_eq_(C)] and with C—H = 0.95 Å. H atoms bonded to O were freely refined.

### Crystal data

**Table:**

C_21_H_16_N_2_O_2_	*F*(000) = 688
*M**_r_* = 328.36	*D*_x_ = 1.322 Mg m^−^^3^
Monoclinic, *P*2_1_/*c*	Melting point: 473 K
Hall symbol: -P 2ybc	Mo *K*α radiation, λ = 0.71073 Å
*a* = 9.7034 (8) Å	Cell parameters from 10768 reflections
*b* = 11.7407 (9) Å	θ = 3.6–27.6°
*c* = 14.9486 (14) Å	µ = 0.09 mm^−^^1^
β = 104.294 (7)°	*T* = 173 K
*V* = 1650.3 (2) Å^3^	Block, colourless
*Z* = 4	0.48 × 0.46 × 0.46 mm

### Data collection

**Table:**

Stoe IPDSII two-circle diffractometer	3235 reflections with *I* > 2σ(*I*)
Radiation source: fine-focus sealed tube	*R*_int_ = 0.034
graphite	θ_max_ = 27.6°, θ_min_ = 3.6°
ω scans	*h* = −12→12
12165 measured reflections	*k* = −13→15
3799 independent reflections	*l* = −18→19

### Refinement

**Table:**

Refinement on *F*^2^	Secondary atom site location: difference Fourier map
Least-squares matrix: full	Hydrogen site location: inferred from neighbouring sites
*R*[*F*^2^ > 2σ(*F*^2^)] = 0.039	H atoms treated by a mixture of independent and constrained refinement
*wR*(*F*^2^) = 0.101	*w* = 1/[σ^2^(*F*_o_^2^) + (0.0529*P*)^2^ + 0.3119*P*] where *P* = (*F*_o_^2^ + 2*F*_c_^2^)/3
*S* = 1.02	(Δ/σ)_max_ < 0.001
3799 reflections	Δρ_max_ = 0.24 e Å^−^^3^
235 parameters	Δρ_min_ = −0.15 e Å^−^^3^
0 restraints	Extinction correction: *SHELXL97* (Sheldrick, 2008), Fc^*^=kFc[1+0.001xFc^2^λ^3^/sin(2θ)]^-1/4^
Primary atom site location: structure-invariant direct methods	Extinction coefficient: 0.030 (2)

### Special details

**Table:**

Geometry. All e.s.d.'s (except the e.s.d. in the dihedral angle between two l.s. planes) are estimated using the full covariance matrix. The cell e.s.d.'s are taken into account individually in the estimation of e.s.d.'s in distances, angles and torsion angles; correlations between e.s.d.'s in cell parameters are only used when they are defined by crystal symmetry. An approximate (isotropic) treatment of cell e.s.d.'s is used for estimating e.s.d.'s involving l.s. planes.
Refinement. Refinement of *F*^2^ against ALL reflections. The weighted *R*-factor *wR* and goodness of fit *S* are based on *F*^2^, conventional *R*-factors *R* are based on *F*, with *F* set to zero for negative *F*^2^. The threshold expression of *F*^2^ > σ(*F*^2^) is used only for calculating *R*-factors(gt) *etc*. and is not relevant to the choice of reflections for refinement. *R*-factors based on *F*^2^ are statistically about twice as large as those based on *F*, and *R*- factors based on ALL data will be even larger.

### Fractional atomic coordinates and isotropic or equivalent isotropic displacement parameters (Å^2^)

**Table:**

	*x*	*y*	*z*	*U*_iso_*/*U*_eq_	
N1	0.72854 (9)	0.56525 (9)	0.70176 (6)	0.0273 (2)	
N2	0.60684 (9)	0.61144 (9)	0.64778 (6)	0.0277 (2)	
O1	0.34395 (9)	0.66647 (10)	0.57881 (6)	0.0434 (3)	
H1	0.428 (2)	0.6418 (18)	0.6211 (13)	0.068 (6)*	
O2	1.07930 (9)	0.61561 (9)	0.60415 (7)	0.0416 (2)	
H2	1.170 (2)	0.6300 (17)	0.5950 (13)	0.073 (6)*	
C3	0.63161 (11)	0.62529 (9)	0.56362 (7)	0.0250 (2)	
C4	0.76965 (11)	0.58763 (10)	0.56388 (7)	0.0267 (2)	
H4	0.8130	0.5885	0.5134	0.032*	
C5	0.82919 (11)	0.54909 (9)	0.65248 (7)	0.0260 (2)	
C11	0.73784 (12)	0.55181 (10)	0.79885 (7)	0.0293 (2)	
C12	0.84714 (13)	0.60411 (11)	0.86344 (8)	0.0354 (3)	
H12	0.9170	0.6479	0.8442	0.042*	
C13	0.85265 (15)	0.59125 (13)	0.95705 (9)	0.0447 (3)	
H13	0.9274	0.6257	1.0021	0.054*	
C14	0.74906 (17)	0.52810 (14)	0.98457 (9)	0.0479 (4)	
H14	0.7532	0.5197	1.0484	0.057*	
C15	0.63982 (16)	0.47744 (13)	0.91924 (9)	0.0437 (3)	
H15	0.5689	0.4349	0.9384	0.052*	
C16	0.63368 (13)	0.48862 (11)	0.82558 (8)	0.0349 (3)	
H16	0.5593	0.4535	0.7806	0.042*	
C31	0.51991 (11)	0.67507 (9)	0.48859 (7)	0.0260 (2)	
C32	0.38098 (12)	0.69291 (11)	0.49811 (8)	0.0308 (2)	
C33	0.27648 (13)	0.73925 (12)	0.42651 (9)	0.0399 (3)	
H33	0.1826	0.7492	0.4336	0.048*	
C34	0.30899 (14)	0.77093 (12)	0.34478 (9)	0.0409 (3)	
H34	0.2378	0.8035	0.2962	0.049*	
C35	0.44618 (15)	0.75502 (12)	0.33381 (8)	0.0395 (3)	
H35	0.4687	0.7765	0.2778	0.047*	
C36	0.54978 (13)	0.70771 (11)	0.40505 (8)	0.0329 (3)	
H36	0.6431	0.6972	0.3971	0.040*	
C51	0.96705 (11)	0.49298 (10)	0.69189 (7)	0.0270 (2)	
C52	1.08989 (12)	0.52664 (10)	0.66442 (8)	0.0304 (2)	
C53	1.21876 (13)	0.46994 (11)	0.69982 (9)	0.0368 (3)	
H53	1.3013	0.4927	0.6811	0.044*	
C54	1.22731 (13)	0.38090 (11)	0.76202 (9)	0.0385 (3)	
H54	1.3155	0.3433	0.7856	0.046*	
C55	1.10706 (14)	0.34660 (11)	0.78991 (8)	0.0364 (3)	
H55	1.1127	0.2858	0.8326	0.044*	
C56	0.97871 (13)	0.40228 (10)	0.75474 (8)	0.0317 (3)	
H56	0.8967	0.3784	0.7737	0.038*	

### Atomic displacement parameters (Å^2^)

**Table:**

	*U*^11^	*U*^22^	*U*^33^	*U*^12^	*U*^13^	*U*^23^
N1	0.0242 (4)	0.0347 (5)	0.0222 (4)	0.0020 (4)	0.0044 (3)	0.0012 (4)
N2	0.0238 (4)	0.0358 (5)	0.0225 (4)	0.0015 (4)	0.0037 (3)	0.0012 (4)
O1	0.0252 (4)	0.0723 (7)	0.0332 (5)	0.0042 (4)	0.0082 (4)	0.0121 (4)
O2	0.0270 (4)	0.0485 (6)	0.0511 (5)	0.0055 (4)	0.0130 (4)	0.0173 (4)
C3	0.0258 (5)	0.0271 (5)	0.0216 (5)	−0.0035 (4)	0.0047 (4)	−0.0019 (4)
C4	0.0270 (5)	0.0299 (6)	0.0235 (5)	−0.0019 (4)	0.0070 (4)	−0.0021 (4)
C5	0.0248 (5)	0.0268 (5)	0.0266 (5)	−0.0013 (4)	0.0066 (4)	−0.0027 (4)
C11	0.0312 (5)	0.0343 (6)	0.0222 (5)	0.0058 (5)	0.0065 (4)	0.0023 (4)
C12	0.0339 (6)	0.0421 (7)	0.0283 (6)	0.0023 (5)	0.0041 (5)	−0.0004 (5)
C13	0.0484 (7)	0.0549 (9)	0.0262 (6)	0.0066 (6)	0.0002 (5)	−0.0023 (6)
C14	0.0656 (9)	0.0546 (9)	0.0243 (6)	0.0107 (7)	0.0127 (6)	0.0059 (6)
C15	0.0552 (8)	0.0448 (8)	0.0359 (7)	0.0039 (6)	0.0201 (6)	0.0090 (6)
C16	0.0371 (6)	0.0373 (6)	0.0313 (6)	0.0017 (5)	0.0102 (5)	0.0028 (5)
C31	0.0272 (5)	0.0260 (5)	0.0228 (5)	−0.0037 (4)	0.0026 (4)	−0.0019 (4)
C32	0.0279 (5)	0.0354 (6)	0.0276 (5)	−0.0033 (4)	0.0038 (4)	0.0011 (5)
C33	0.0293 (6)	0.0458 (7)	0.0394 (7)	0.0004 (5)	−0.0013 (5)	0.0041 (6)
C34	0.0420 (7)	0.0379 (7)	0.0337 (6)	−0.0039 (5)	−0.0079 (5)	0.0066 (5)
C35	0.0506 (7)	0.0385 (7)	0.0261 (5)	−0.0070 (6)	0.0033 (5)	0.0049 (5)
C36	0.0367 (6)	0.0355 (6)	0.0263 (5)	−0.0038 (5)	0.0071 (4)	0.0005 (5)
C51	0.0267 (5)	0.0276 (5)	0.0256 (5)	0.0017 (4)	0.0040 (4)	−0.0026 (4)
C52	0.0285 (5)	0.0316 (6)	0.0307 (5)	0.0026 (4)	0.0066 (4)	0.0000 (5)
C53	0.0280 (6)	0.0393 (7)	0.0422 (7)	0.0052 (5)	0.0072 (5)	−0.0010 (5)
C54	0.0347 (6)	0.0360 (7)	0.0411 (7)	0.0106 (5)	0.0024 (5)	−0.0016 (5)
C55	0.0443 (7)	0.0297 (6)	0.0326 (6)	0.0071 (5)	0.0048 (5)	0.0013 (5)
C56	0.0357 (6)	0.0297 (6)	0.0296 (5)	0.0003 (5)	0.0078 (5)	−0.0020 (5)

### Geometric parameters (Å, °)

**Table:**

N1—N2	1.3672 (13)	C15—H15	0.9500
N1—C5	1.3740 (13)	C16—H16	0.9500
N1—C11	1.4403 (13)	C31—C36	1.4033 (15)
N2—C3	1.3477 (13)	C31—C32	1.4057 (15)
O1—C32	1.3765 (14)	C32—C33	1.3907 (17)
O1—H1	0.947 (19)	C33—C34	1.3859 (18)
O2—C52	1.3666 (15)	C33—H33	0.9500
O2—H2	0.94 (2)	C34—C35	1.3935 (19)
C3—C4	1.4097 (15)	C34—H34	0.9500
C3—C31	1.4744 (15)	C35—C36	1.3873 (17)
C4—C5	1.3838 (15)	C35—H35	0.9500
C4—H4	0.9500	C36—H36	0.9500
C5—C51	1.4769 (15)	C51—C56	1.4060 (16)
C11—C12	1.3887 (17)	C51—C52	1.4098 (15)
C11—C16	1.3896 (16)	C52—C53	1.4000 (16)
C12—C13	1.3953 (17)	C53—C54	1.3878 (19)
C12—H12	0.9500	C53—H53	0.9500
C13—C14	1.391 (2)	C54—C55	1.3921 (19)
C13—H13	0.9500	C54—H54	0.9500
C14—C15	1.386 (2)	C55—C56	1.3898 (17)
C14—H14	0.9500	C55—H55	0.9500
C15—C16	1.3928 (17)	C56—H56	0.9500
			
N2—N1—C5	111.28 (8)	C32—C31—C3	121.69 (9)
N2—N1—C11	117.92 (8)	O1—C32—C33	117.66 (11)
C5—N1—C11	130.48 (9)	O1—C32—C31	121.29 (10)
C3—N2—N1	105.77 (8)	C33—C32—C31	121.04 (11)
C32—O1—H1	106.6 (11)	C34—C33—C32	120.13 (12)
C52—O2—H2	108.2 (12)	C34—C33—H33	119.9
N2—C3—C4	110.36 (9)	C32—C33—H33	119.9
N2—C3—C31	119.40 (9)	C33—C34—C35	119.98 (11)
C4—C3—C31	130.24 (9)	C33—C34—H34	120.0
C5—C4—C3	106.08 (9)	C35—C34—H34	120.0
C5—C4—H4	127.0	C36—C35—C34	119.72 (11)
C3—C4—H4	127.0	C36—C35—H35	120.1
N1—C5—C4	106.50 (9)	C34—C35—H35	120.1
N1—C5—C51	122.78 (9)	C35—C36—C31	121.51 (11)
C4—C5—C51	130.59 (9)	C35—C36—H36	119.2
C12—C11—C16	121.40 (11)	C31—C36—H36	119.2
C12—C11—N1	119.95 (10)	C56—C51—C52	118.20 (10)
C16—C11—N1	118.63 (10)	C56—C51—C5	121.32 (10)
C11—C12—C13	118.88 (12)	C52—C51—C5	120.43 (10)
C11—C12—H12	120.6	O2—C52—C53	121.84 (10)
C13—C12—H12	120.6	O2—C52—C51	118.35 (10)
C14—C13—C12	120.19 (13)	C53—C52—C51	119.81 (11)
C14—C13—H13	119.9	C54—C53—C52	120.77 (11)
C12—C13—H13	119.9	C54—C53—H53	119.6
C15—C14—C13	120.23 (12)	C52—C53—H53	119.6
C15—C14—H14	119.9	C53—C54—C55	120.18 (11)
C13—C14—H14	119.9	C53—C54—H54	119.9
C14—C15—C16	120.23 (13)	C55—C54—H54	119.9
C14—C15—H15	119.9	C56—C55—C54	119.32 (12)
C16—C15—H15	119.9	C56—C55—H55	120.3
C11—C16—C15	119.06 (12)	C54—C55—H55	120.3
C11—C16—H16	120.5	C55—C56—C51	121.72 (11)
C15—C16—H16	120.5	C55—C56—H56	119.1
C36—C31—C32	117.60 (10)	C51—C56—H56	119.1
C36—C31—C3	120.71 (10)		
			
C5—N1—N2—C3	−0.45 (12)	C4—C3—C31—C32	170.94 (11)
C11—N1—N2—C3	173.77 (10)	C36—C31—C32—O1	−178.03 (11)
N1—N2—C3—C4	0.08 (12)	C3—C31—C32—O1	1.20 (17)
N1—N2—C3—C31	−179.44 (9)	C36—C31—C32—C33	1.20 (18)
N2—C3—C4—C5	0.30 (13)	C3—C31—C32—C33	−179.57 (12)
C31—C3—C4—C5	179.76 (11)	O1—C32—C33—C34	177.90 (12)
N2—N1—C5—C4	0.63 (13)	C31—C32—C33—C34	−1.4 (2)
C11—N1—C5—C4	−172.65 (11)	C32—C33—C34—C35	0.8 (2)
N2—N1—C5—C51	−175.58 (10)	C33—C34—C35—C36	−0.2 (2)
C11—N1—C5—C51	11.14 (19)	C34—C35—C36—C31	0.0 (2)
C3—C4—C5—N1	−0.55 (12)	C32—C31—C36—C35	−0.55 (18)
C3—C4—C5—C51	175.26 (11)	C3—C31—C36—C35	−179.78 (11)
N2—N1—C11—C12	−121.61 (12)	N1—C5—C51—C56	35.52 (16)
C5—N1—C11—C12	51.30 (18)	C4—C5—C51—C56	−139.69 (13)
N2—N1—C11—C16	56.64 (15)	N1—C5—C51—C52	−146.91 (11)
C5—N1—C11—C16	−130.45 (13)	C4—C5—C51—C52	37.88 (18)
C16—C11—C12—C13	0.84 (19)	C56—C51—C52—O2	−179.27 (11)
N1—C11—C12—C13	179.04 (11)	C5—C51—C52—O2	3.09 (16)
C11—C12—C13—C14	−0.8 (2)	C56—C51—C52—C53	−0.05 (17)
C12—C13—C14—C15	0.1 (2)	C5—C51—C52—C53	−177.69 (10)
C13—C14—C15—C16	0.5 (2)	O2—C52—C53—C54	179.03 (12)
C12—C11—C16—C15	−0.23 (18)	C51—C52—C53—C54	−0.17 (18)
N1—C11—C16—C15	−178.46 (11)	C52—C53—C54—C55	0.14 (19)
C14—C15—C16—C11	−0.5 (2)	C53—C54—C55—C56	0.10 (19)
N2—C3—C31—C36	169.56 (11)	C54—C55—C56—C51	−0.32 (18)
C4—C3—C31—C36	−9.85 (18)	C52—C51—C56—C55	0.29 (17)
N2—C3—C31—C32	−9.64 (16)	C5—C51—C56—C55	177.91 (11)

### Hydrogen-bond geometry (Å, °)

**Table:**

*D*—H···*A*	*D*—H	H···*A*	*D*···*A*	*D*—H···*A*
O2—H2···O1^i^	0.94 (2)	1.81 (2)	2.7524 (12)	176.6 (19)
O1—H1···N2	0.947 (19)	1.718 (19)	2.5863 (12)	150.9 (17)

Symmetry codes: (i) *x*+1, *y*, *z*.

## References

[bb1] Ahmad, R., Malik, M. A., Zia-ul-Haq, M., Duddeek, H., Stefaniak, L. & Kowski, J. S. (1997). *Monatsh. Chem.* **128**, 633–640.

[bb2] Beeam, C. F., Hall, H. L., Huff, A. M., Tummons, R. C. & Grady, S. A. O. (1984). *J. Heteroat. Chem.* **21**, 1897–1902.

[bb3] Bonati, F. (1980). *Chim. Ind. (Roma)*, **62**, 323–328.

[bb4] Elguero, J. (1983). *Comprehensive Heterocyclic Chemistry*, Vol. 5, Part 4A, pp. 167 and 304. Elmford, New York: Pergamon Press.

[bb5] Freyer, W. & Radeglia, R. (1981). *Monatsh. Chem.* **112**, 105–117.

[bb6] Sheldrick, G. M. (2008). *Acta Cryst.* A**64**, 112–122. 10.1107/S0108767307043930 18156677

[bb7] Spek, A. L. (2003). *J. Appl. Cryst.* **36**, 7–13.

[bb8] Stoe & Cie (2001). *X-AREA* Stoe & Cie, Darmstadt, Germany.

